# Neural correlates of map representation and spatial orientation in orienteering experts: an fNIRS study

**DOI:** 10.3389/fnhum.2026.1769082

**Published:** 2026-05-21

**Authors:** Long Wang, Shi Jia Ou, Yang Liu

**Affiliations:** 1 Northwestern Polytechnical University, Xi'an, China; 2 Northeast Normal University, Changchun, China; 3 Shaanxi Normal University, Xi'an, China

**Keywords:** functional near-infrared spectroscopy, map representation, orienteering, prefrontal cortex, spatial orientation

## Abstract

This study investigated the neural mechanisms underlying spatial navigation expertise in orienteering using a mixed-factorial design with skill level (expert vs. novice) as a between-subjects factor and map orientation (normal vs. rotated) as a within-subjects factor. Forty-eight orienteers participated in the experiment. Functional near-infrared spectroscopy (fNIRS) was used to assess behavioral performance and hemodynamic responses in the prefrontal cortex (PFC). Behavioral results showed that experts achieved higher accuracy than novices, whereas map rotation reduced accuracy across participants. A significant interaction was observed for reaction time: Experts responded significantly faster than novices, specifically in the rotated condition. Neuroimaging results showed that the rotated condition elicited greater activation in the right ventrolateral prefrontal cortex (R-VLPFC). In contrast, novices showed greater activation in the left dorsolateral prefrontal cortex (L-DLPFC), whereas experts exhibited attenuated L-DLPFC activation under rotated-orientation conditions. In addition, the frontal pole showed significant group differences across conditions. Accuracy in the expert group was negatively correlated with dorsolateral prefrontal cortex activation. Together, these findings suggest that expert orienteers may exhibit a neural-efficiency advantage, characterized by superior performance with reduced cortical recruitment, and highlight the R-VLPFC as an important region involved in spatial reorientation.

## 1 Introduction

In daily life, people need to navigate through complex spatial environments. Successful completion of wayfinding tasks fundamentally depends on accurate map representation, which critically influences navigation behavior (Waddington and Heisz, 2023; Ye, 2010). Orienteering, as a competitive sport requiring sequential visits to control points using maps and compasses, relies heavily on map representation, which refers to the cognitive process by which orienteers match map symbols with actual terrain features (Mottet and Saury, 2013; Newton and Holmes, 2017).

During this process, orienteers must analyze map information based on their existing knowledge and experience, which involves the extraction and integration of salient features of map symbols to construct a three-dimensional representation of the terrain from a two-dimensional map and subsequently align this mental model with real-world environmental information for target identification (Guo L. M. et al., 2023; Kolb et al., 1987; Liu and He, 2016). It essentially constitutes an information processing mechanism bridging cartographic symbols and environmental perception. Concurrently, during orienteering competitions, continuous changes in movement direction and spatial positioning create inconsistencies between map representation and actual spatial orientation (Fang, 2019). Orienteers must recalibrate the spatial correspondence between the map and environmental landmarks and use their brains to reformulate map representation and construct updated cognitive maps. Such dynamic spatial updating requires superior spatial orientation abilities (Uttal et al., 2013). Eccles et al. (2002) applied grounded theory to identify three core factors that influence orienteering performance: the orienteering map, real-world environmental information, and visual attention allocation during navigation (Liu and He, 2021). The effectiveness of map representation is affected by cognitive load, which in turn affects the efficacy of route decision-making. The ability for spatial orientation plays a crucial role in map recognition and shows a strong correlation with task performance (Song et al., 2021; Yi, 2022). As a result, different spatial orientations may influence orienteers’ performance in map-representation tasks. Investigating the cognitive processes behind this phenomenon holds practical value for optimizing orienteering training and developing structured practice strategies to improve wayfinding skills. Elite orienteering athletes tend to form more precise spatial representations, linking high-level cognitive performance with neural structures involved in cognition and navigation.

A key characteristic of cognitive maps is their reliance on multiple types of spatial memory, including survey knowledge (relationships between landmarks) and route knowledge (paths learned through experience) (Liu and Kan, 2024). Researchers have used eye-tracking and fNIRS technologies to explore the cognitive characteristics of expert and novice orienteers from different perspectives, such as visual attention, working memory (Liu, 2021), and cognitive load (Song et al., 2021). These studies have demonstrated that expert orienteers outperform novices in domain-specific tasks, characterized by more efficient visual search and greater memory capacity. Moreover, orienteering training has been shown to positively affect cognitive abilities among different populations, including middle school students (Tang, 2023), children with ADHD (Bao et al., 2021), and older adults (Biehl-Printes et al., 2024; Zheng, 2014). To uncover the neural mechanisms driving these cognitive improvements, researchers have investigated specialized cognitive processes in orienteering, such as spatial memory (Liu et al., 2022), route selection strategies (Zhao et al., 2022), mental rotation abilities (Yi, 2022), and spatial distance perception (Tang and Liu, 2021). The findings indicate that different orienteering tasks elicit distinct activation patterns in subregions of the prefrontal cortex (PFC), reflecting training effects. Existing studies suggest that the prefrontal cortex is broadly involved in executive control, working memory, and spatial transformation during orienteering-related tasks. More specifically, the dorsolateral prefrontal cortex (DLPFC) has consistently been associated with the maintenance and manipulation of task-relevant information, performance monitoring, and executive control under increased task demands (Friedman and Robbins, 2022). By contrast, the ventrolateral prefrontal cortex (VLPFC) has been linked more closely to attentional reorientation, interference suppression, and the selection of task-relevant representations when competing spatial information must be resolved (Levy and Wagner, 2011). These functional distinctions are particularly relevant to map-representation tasks in orienteering, as rotated-map processing requires participants to suppress an initially mismatched spatial reference, re-establish correspondence between the map and the environment, and update spatial representations online (Guo H. et al., 2023). However, the specific roles of prefrontal subregions during map representation under different spatial orientations remain insufficiently defined. In particular, it remains unclear whether the VLPFC primarily supports the core process of spatial reorientation during rotated-map processing, whereas the DLPFC reflects additional working-memory and executive-control demands, particularly in novice orienteers. Moreover, little is known about whether these subregional activation patterns differ systematically between expert and novice orienteers.

Functional near-infrared spectroscopy (fNIRS) is a useful neuroimaging modality, distinguished by its ecological validity—particularly its portability, non-invasive nature, and robustness to motion artifacts relative to traditional techniques (Pinti et al., 2020; Yücel et al., 2017). Consequently, this technology has been widely used to investigate cortical dynamics in sports neuroscience (Ferrari and Quaresima, 2012; Kopton and Kenning, 2014). By quantifying hemodynamic responses, reflected in concentration changes in oxygenated hemoglobin (HbO_2_) and deoxygenated hemoglobin (HbR), fNIRS provides a reliable surrogate measure of neural activity. Spatial navigation and cognitive map formation are often linked to medial temporal structures, particularly the hippocampal system. However, the present study focuses on map representation under changing spatial orientations, a process that places substantial demands on online executive control, working memory, attentional allocation, interference suppression, and spatial transformation (Mandrick et al., 2013; Miller and Cohen, 2001). These functions are more directly associated with the prefrontal cortex than with medial temporal regions. In addition, because fNIRS is primarily sensitive to cortical surface signals, the PFC represents a methodologically suitable and theoretically relevant region of interest for examining the neural dynamics of map-based spatial reorientation in orienteers. In summary, this research investigates the cognitive characteristics of map representation in expert and novice orienteers through orienteering-specific tasks, using image-based stimuli under two conditions—standard orientation and rotated orientation. Based on this framework, the present study proposed the following hypotheses. First, expert orienteers would demonstrate better behavioral performance than novices, as reflected by higher accuracy and shorter reaction times, particularly under the rotated-orientation condition. Second, compared with the normal-orientation condition, the rotated-orientation condition would elicit greater activation in the right ventrolateral prefrontal cortex (R-VLPFC) in both groups, because map rotation requires spatial reorientation, interference suppression, and the updating of map-to-environment correspondence. Third, novice orienteers would show greater activation in the left dorsolateral prefrontal cortex (L-DLPFC) than experts under the rotated-orientation condition, reflecting higher working-memory and executive-control demands, whereas experts would exhibit relatively attenuated activation consistent with greater neural efficiency.

## 2 Materials and methods

### 2.1 Participants

The study cohort comprised 48 athletes who were stratified into 2 distinct groups based on their competitive proficiency and training history. The expert cohort included 17 individuals (mean training tenure: 8.70 ± 2.75 years) classified as National First-Level Athletes, distinguished by top-six placements in either the National Orienteering Championships or the National Orienteering Elite Competition. Conversely, the novice cohort consisted of the remaining 31 university-level orienteers, with an average experience of 2.00 ± 0.48 years. To ensure sample homogeneity, eligibility was restricted to participants meeting specific inclusion criteria: (1) right-handed dominance with normal or corrected-to-normal visual acuity; (2) absence of neurological, psychiatric, or cardiovascular pathologies, as well as no history of traumatic brain injury; and (3) computer literacy and no prior exposure to the experimental protocol. A briefing session was conducted 24 h prior to data collection to outline procedural details. Participants were strictly instructed to obtain sufficient sleep and maintain scalp hygiene to facilitate optimal signal acquisition. In addition, all methods used in this study were conducted in accordance with relevant guidelines and regulations, including the Declaration of Helsinki, and were approved by the Ethics Committee of Shaanxi Normal University (Approval number: 202416006). Written informed consent was obtained from all participants.

### 2.2 Experimental design and materials

We implemented a mixed experimental design with expertise (expert and novice) serving as the between-group variable and spatial alignment (normal and rotated) as the within-group variable. Dependent measures included behavioral performance (accuracy and reaction time) and cortical activation (HbO_2_ levels in the PFC) recorded via fNIRS.

**Figure 1:**
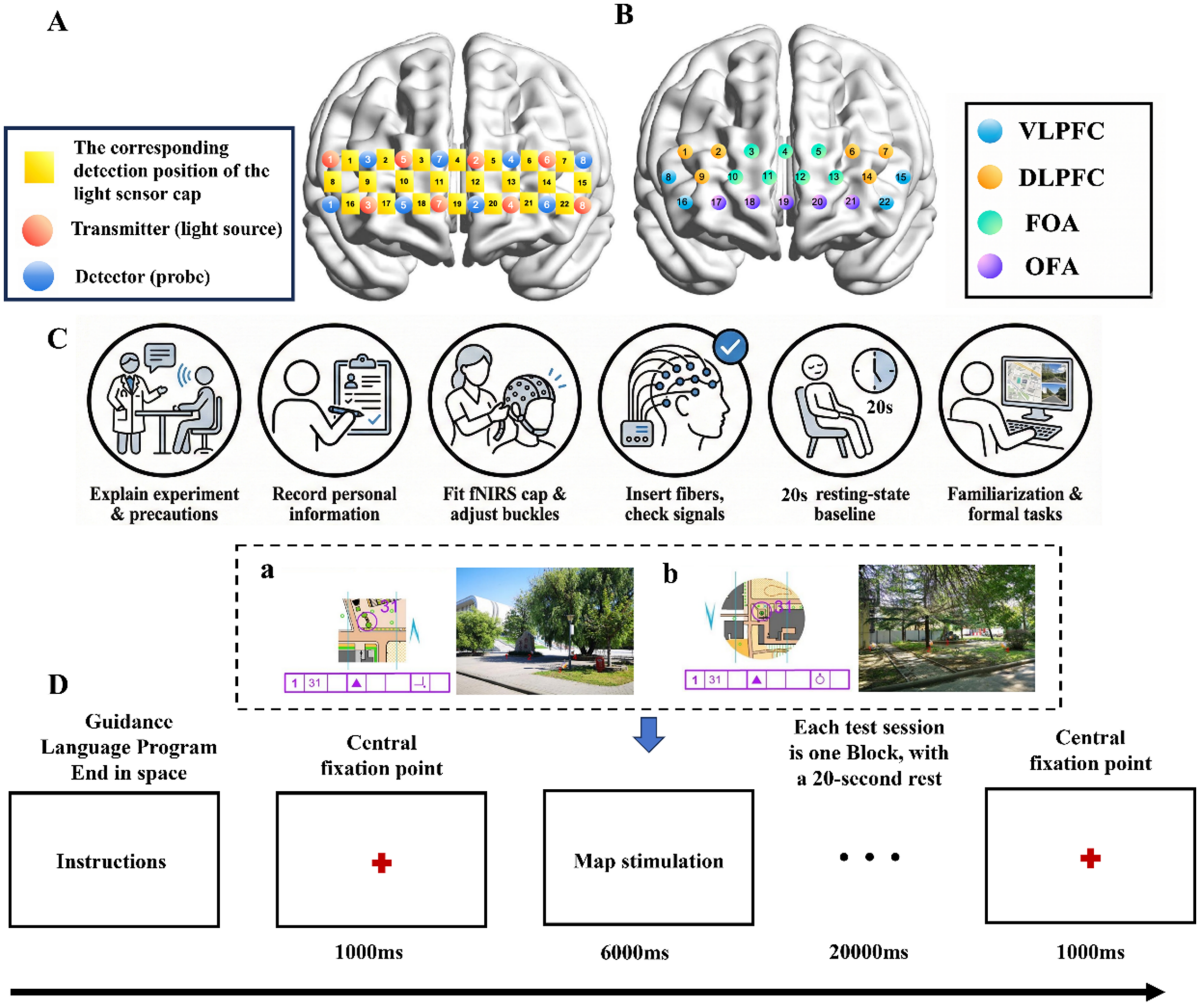
Probe placement schematic and experimental procedure flowchart. **(a)** Normal orientation and **(b)** rotation orientation. **(A)** The corresponding detection positions and channels of the photo receptor cap in the prefrontal cortex region. **(B)** The corresponding detection area of the photo receptor capillary. **(C)** Experimental test flowchart. **(D)** Map recognition task flowchart.

Experimental stimuli consisted of four components presented simultaneously on each trial: (1) an orienteering map, (2) a control-point descriptor, (3) a compass indicator, and (4) a real-world scene photograph. As shown in Figure 1, the control-point descriptor was presented beneath the map and included the target control-point number, along with symbolic information indicating the target location relative to a mapped terrain feature. The compass indicator was presented as a directional arrow indicating north and served as a reference for judging the orientation relationship between the map and the scene. The photograph of the real-world scene depicted the terrain corresponding to the orienteering map. Participants were required to integrate the map, the control-point descriptor, and the directional cue in order to identify the target location in the scene photograph. The task included two spatial-orientation conditions. In the normal-orientation condition, the map was aligned with the environmental reference frame shown in the scene photograph. In the rotated-orientation condition, the map was rotated relative to the environmental reference frame, whereas the scene photograph remained unchanged. The rotated maps were presented at 90°, 180°, or 270° relative to the correct orientation, and these rotation angles varied across trials in the rotated condition. In each scene photograph, four candidate target locations were marked with flags, and only one location correctly matched the control point indicated on the map. As shown in Figure 1A, a sample trial involved locating point 31 (described as a rock’s southeast corner). In this instance, the compass confirmed the map’s orientation, identifying option 2 as the correct target. To guarantee ecological validity, all materials underwent rigorous verification by three professional orienteers.

### 2.3 Measuring instruments

Hemodynamic signals were recorded using a portable triple-wavelength fNIRS system (LIGHTNIRS, Shimadzu Corporation, Japan), operating at an emission spectrum of 780, 805, and 830 nm with a temporal resolution of 13.33 Hz. While the system monitors total, oxygenated, and deoxygenated hemoglobin, oxygenated hemoglobin (HbO_2_) was selected as the primary biomarker for neural activation. This decision was based on its superior sensitivity to cerebral blood flow variations and higher signal-to-noise ratio than deoxygenated hemoglobin (HbR) (Hoshi, 2003; Huppert et al., 2006). The optical probe assembly was positioned over the prefrontal cortex (PFC), aligning with the Fp1–Fp2 axis of the international 10–20 system. The montage consisted of two 2 × 8 optode arrays, creating a total of 22 measurement channels with a fixed inter-optode separation of 3 cm. To ensure anatomical precision, the 3D coordinates of all optodes were digitized using a FASTRAK system. These coordinates were subsequently registered to the Montreal Neurological Institute (MNI) space using the probabilistic registration algorithm in NIRS_SPM, enabling identification of corresponding Brodmann areas (see Figures 1A,B).

### 2.4 Procedure

Prior to the experiment, participants were acquainted with the laboratory environment, provided personal data for recording, and briefed on all necessary experimental precautions. Following this, the experimenters securely placed the fNIRS cap on the participants’ heads, adjusted the nylon buckles to ensure a proper fit and connection, and positioned the participants for calibration. Optical fibers were then gently inserted into each probe, followed by a light transmission and reception check. Once signal stability for all channels was confirmed, a baseline (zero-point) correction was performed, and data acquisition began. The subsequent testing involved two main tasks: the normal orientation task and the rotating orientation task, both following identical operational procedures (see Figure 1C).

Stimulus presentation and data collection were orchestrated using E-Prime 3.0 (Psychology Software Tools, Pittsburgh, PA). The protocol consisted of a preliminary familiarization phase followed by the formal data collection block. Each session commenced with onscreen instructional text; participants were required to comprehend the task requirements before initiating the sequence via the spacebar.

Individual trials began with a central fixation cross being displayed for 1,000 ms, which was immediately replaced by the target stimulus. During the critical task phase, participants were instructed to determine which of the four flagged locations in the scene photograph matched the target control point using the map, the control-point descriptor, and compass information. Participants responded by pressing the corresponding numerical key (1–4). Each trial had a response window of 6,000 ms. In the practice phase, immediate feedback was provided after each response, whereas no feedback was presented during the formal experiment. Trials without a response within 6,000 ms were recorded as missed responses. The schematic flow of the experiment is detailed in Figure 1D.

The experimental session commenced with a 30-s resting-state recording to establish a physiological baseline, during which participants were instructed to remain relaxed and quiescent. The subsequent task adhered to the same protocol as the familiarization phase, with the notable exception that performance feedback was withheld.

We used a block-design paradigm with six blocks, each containing 4 trials, for a total of 24 trials. The 24 trials were evenly divided between the normal-orientation (12 trials) and rotated-orientation conditions (12 trials). The trial order was randomized for each participant. A 20-s rest interval was inserted between blocks to allow the hemodynamic response to return to baseline. This pause was designed to facilitate hemodynamic recovery in the prefrontal cortex (PFC), ensuring that oxygenation levels returned to baseline before the next block. Throughout the session, behavioral metrics (reaction time, accuracy) and cerebral hemodynamic data were logged synchronously by the stimulus software and the NIRS system, respectively. Given the high cognitive demands of the task, the number of trials per block and the inter-block rest interval were designed to reduce fatigue and to allow the hemodynamic response to return to baseline before the next block.

### 2.5 Data analysis

#### 2.5.1 Statistical analysis

Statistical procedures were initiated by confirming that all measurement variables met the criteria for normal distribution (*p* > 0.05). The primary analysis used a repeated-measures ANOVA, with group as a between-subjects factor, to evaluate the main and interaction effects of skill level and spatial orientation. Where significant interactions emerged, simple main effects were analyzed using the Bonferroni adjustment for multiple comparisons. All statistical tests were two-tailed, with significance determined by a *p*-value of less than 0.05.

#### 2.5.2 fNIRS data preprocessing

Data analysis was performed using the NIRS_SPM toolbox within the MATLAB R2013b environment. Initially, raw light-intensity signals were converted into optical density and subsequently into concentration changes in oxygenated hemoglobin (HbO_2_) and deoxygenated hemoglobin (HbR). Although both HbO_2_ and HbR were derived during preprocessing, the present study focused on HbO_2_ as the primary hemodynamic index in the main statistical analyses. This decision was based on previous fNIRS studies showing that HbO_2_ generally exhibits a larger response amplitude, higher sensitivity to task-related cerebral blood flow changes, and a higher signal-to-noise ratio than HbR in cognitive activation paradigms (Kamran et al., 2016; Yang et al., 2020). HbR was therefore not included as a primary outcome measure in the present study.

Channels with poor signal quality were identified using the signal-to-noise ratio criterion implemented in NIRS_SPM and excluded from further analysis. To reduce low-frequency drift, signal detrending was performed using the discrete cosine transform (DCT) approach implemented in NIRS_SPM. Temporal smoothing was then applied using the canonical hemodynamic response function (HRF) model provided in the toolbox.

For first-level analysis, a general linear model (GLM) was constructed for each participant. The normal-orientation and rotated-orientation conditions were modeled as separate regressors based on block onset and duration, and each regressor was convolved with the canonical HRF. Beta coefficients (*β*-values) were estimated for each channel and condition and used as indices of task-related cortical activation, with higher values denoting increased neural engagement.

At the group level, β-values were submitted to a 2 × 2 mixed-design ANOVA with group (expert vs. novice) as the between-subjects factor and orientation (normal vs. rotated) as the within-subjects factor. Because channel-wise analyses were conducted across 22 fNIRS channels, *p*-values were corrected for multiple comparisons using the false discovery rate (FDR) procedure. FDR correction was applied separately to the *p*-values for the main effect of group, the main effect of orientation, and the group × orientation interaction across all channels. In cases where the assumption of sphericity was violated, degrees of freedom were corrected using the Greenhouse–Geisser estimates. Post-hoc comparisons were adjusted using the Bonferroni correction where appropriate (*α* < 0.05). Finally, significant activation patterns were mapped onto a 3D cortical surface using BrainNet Viewer.

## 3 Results

### 3.1 Behavioral results

For accuracy, a significant main effect of group was observed, *F*(1,46) = 38.380, *p* = 0.001, *η*^2^ = 0.593, with the expert group showing significantly higher accuracy than the novice group. A significant main effect of spatial orientation was also found [*F*(1,46) = 36.923, *p* = 0.001, *η*^2^ = 0.440], indicating that accuracy was higher in the normal-orientation condition than in the rotated-orientation condition. The interaction between group and spatial orientation was not significant [*F*(1,46) = 1.236, *p* = 0.272, *η*^2^ = 0.026].

For reaction time, significant main effects were found for both Group, *F*(1,46) = 17.525, *p* = 0.001, *η*^2^ = 0.272, and spatial orientation [*F*(1,46) = 55.099, *p* = 0.001, *η*^2^ = 0.540], indicating that experts responded faster than novices and that responses were faster in the normal-orientation condition than in the rotated-orientation condition. In addition, the group × spatial orientation interaction was significant [*F*(1,46) = 22.801, *p* = 0.001, *η*^2^ = 0.327] (Table 1).

**Table 1:** Behavioral results of map representation (M ± SD).

Group	Accuracy (%)		Reaction time (ms)	
	Normal orientation	Rotated orientation	Normal orientation	Rotated orientation
Expert group	0.78 ± 0.05	0.72 ± 0.03	3669.71 ± 318.41	3825.32 ± 234.83
Novice group	0.65 ± 0.05	0.60 ± 0.07	3726.44 ± 299.80	4443.29 ± 402.47

**Figure 2:**
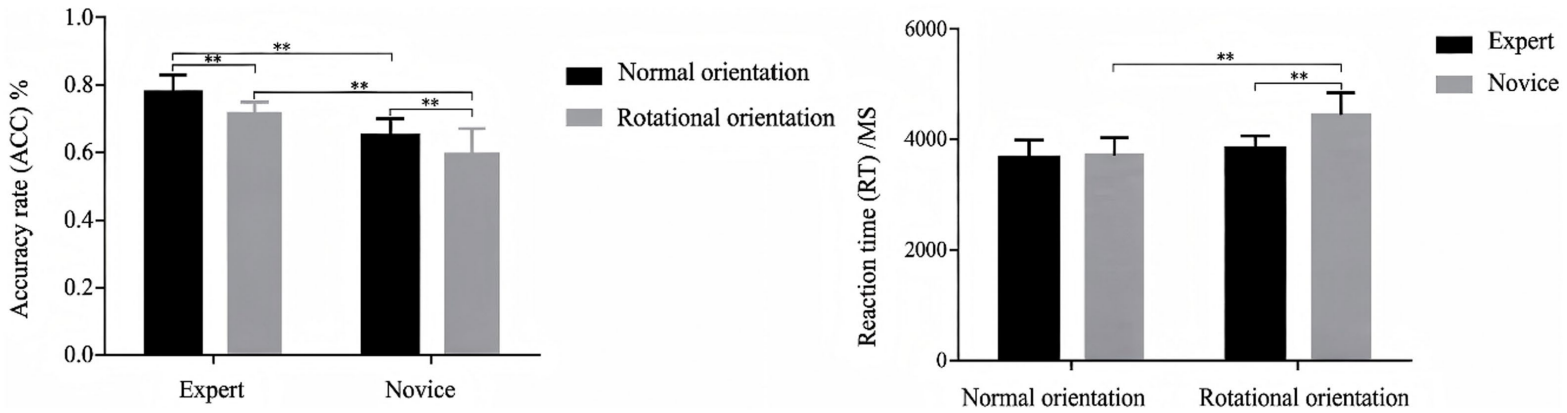
Results of behavioral performance of map representation in orienteers at different skill levels. *p < 0.05, **p < 0.01.

Simple-effects analysis showed that, in the normal-orientation condition, the between-group difference in reaction time was not significant [*F*(1,46) = 0.381, *p* = 0.137, *η*^2^ = 0.046]. In contrast, under the rotated-orientation condition, experts responded significantly faster than novices [*F*(1,46) = 33.363, *p* = 0.001, *η*^2^ = 0.415]. Within the novice group, reaction times were significantly shorter in the normal-orientation condition than in the rotated-orientation condition [*F*(1,46) = 107.215, *p* = 0.001, *η*^2^ = 0.695]. By contrast, no significant difference between the two orientation conditions was observed in the expert group [*F*(1,46) = 0.381, *p* = 0.108, *η*^2^ = 0.054] (see Figure 2).

### 3.2 Blood oxygen activation results

**Figure 3:**
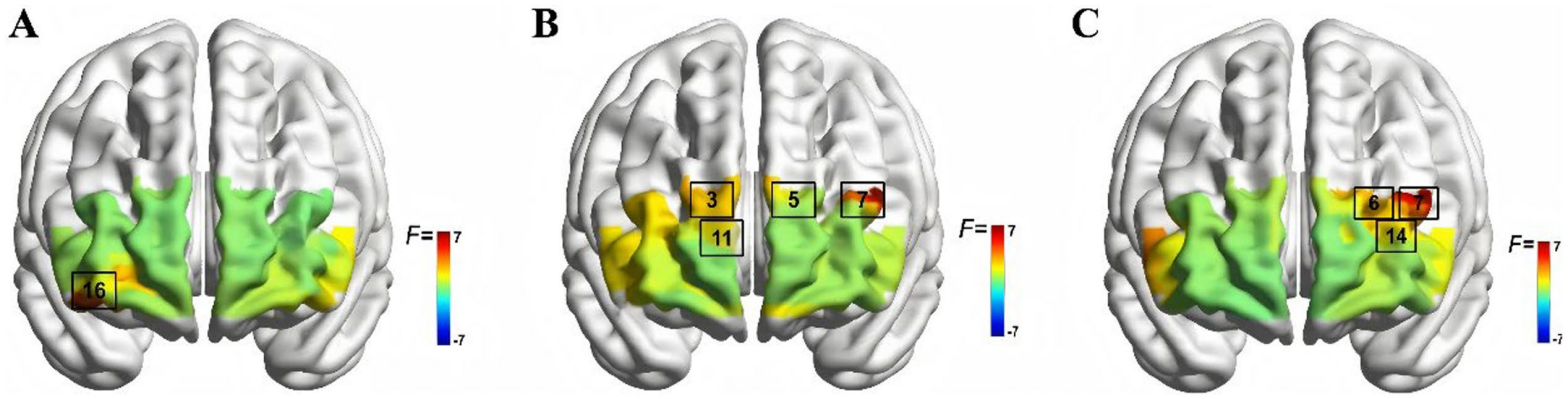
fNIRS-measured brain activation results. **(A)** Spatial orientation main effect, **(B)** group main effect, and **(C)** interaction effect.

After the false discovery rate (FDR) correction for multiple comparisons across the 22 channels, a significant main effect of orientation was observed only in Ch16 [*F*(1,46) = 7.642, *p* = 0.008, *η*^2^ = 0.142], with greater oxygenation activation in the rotated-orientation condition than in the normal-orientation condition. Significant main effects of group were observed in Ch3 [*F*(1,46) = 7.366, *p* = 0.009, *η*^2^ = 0.138], Ch5 [*F*(1,46) = 6.880, *p* = 0.012, *η*^2^ = 0.130], Ch7 [*F*(1,46) = 7.317, *p* = 0.010, *η*^2^ = 0.137], Ch11 [*F*(1,46) = 5.210, *p* = 0.027, *η*^2^ = 0.102], with higher activation in the novice group than in the expert group. In addition, significant group × orientation interactions were found in Ch6 [*F*(1,46) = 5.634, *p* = 0.022, *η*^2^ = 0.109], Ch7 [*F*(1,46) = 7.909, *p* = 0.007, *η*^2^ = 0.147], Ch14 [*F*(1,46) = 5.964, *p* = 0.019, *η*^2^ = 0.115] (see Table 2; Figure 3).

**Table 2:** Mean *β*-values for channels showing significant effects in the prefrontal cortex (M ± SD).

CHID	Expert group		Novice group	
	Normal orientation	Rotational orientation	Normal orientation	Rotational orientation
CH 3	0.0002 ± 0.0037	−0.0009 ± 0.0043	0.0010 ± 0.0029	0.0023 ± 0.0031
CH 5	−0.0001 ± 0.0047	−0.0008 ± 0.0044	0.0003 ± 0.0025	0.0025 ± 0.0029
CH 6	0.0014 ± 0.0032	−0.0004 ± 0.0044	0.0000 ± 0.0020	0.0016 ± 0.0035
CH 7	0.00005 ± 0.0018	−0.0016 ± 0.0034	0.0005 ± 0.0031	0.0020 ± 0.0032
CH 11	−0.0004 ± 0.0039	−0.0004 ± 0.0058	0.0006 ± 0.0027	0.0018 ± 0.0030
CH 14	0.0007 ± 0.0043	−0.0017 ± 0.0057	−0.0001 ± 0.0028	0.0057 ± 0.0017
CH 16	−0.0027 ± 0.0054	0.0007 ± 0.0058	0.0005 ± 0.0048	0.0025 ± 0.0058

**Figure 4:**
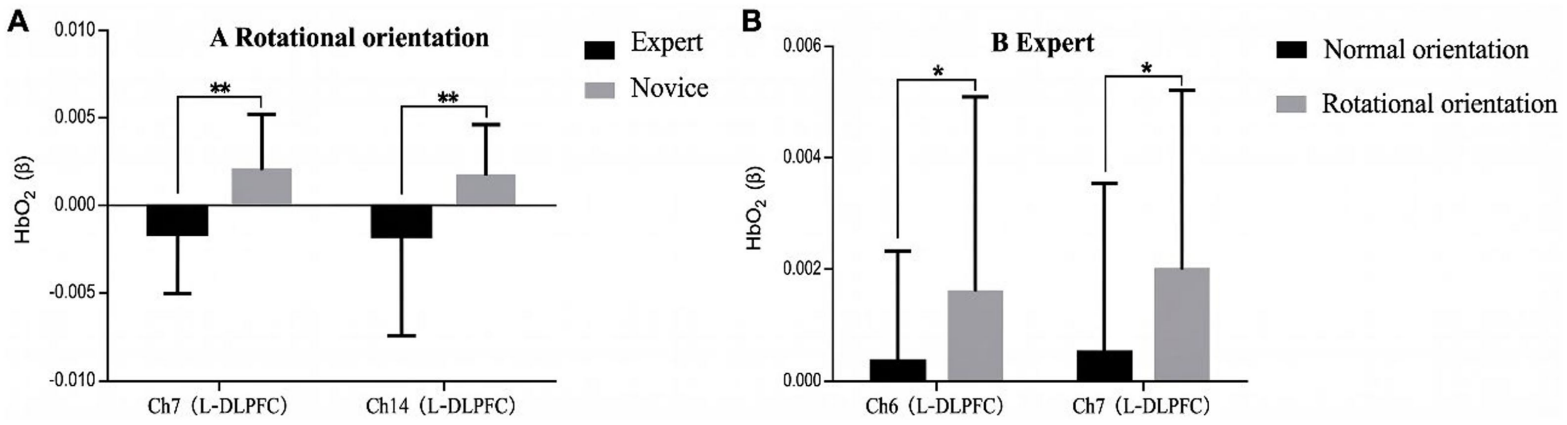
Results of the simple-effects analysis: **(A)** Rotated orientation and **(B)** expert group. *p < 0.05 and **p < 0.01.

Simple-effects analysis showed that, under the normal-orientation condition, no significant between-group differences in oxygenation activation were observed across channels. Under the rotated-orientation condition, however, activation was significantly lower in the expert group than in the novice group at Ch7 [*F*(1,46) = 12.397, *p* = 0.001, *η*^2^ = 0.212] and Ch14 [*F*(1,46) = 7.490, *p* = 0.009, *η*^2^ = 0.140]. No significant between-condition differences were found in the expert group. In contrast, within the novice group, activation was significantly higher in the rotated-orientation condition than in the normal-orientation condition at Ch7 [*F*(1,46) = 5.198, *p* = 0.027, *η*^2^ = 0.102], whereas the difference at Ch6 reached only marginal significance [*F*(1,46) = 3.846, *p* = 0.05, *η*^2^ = 0.077] (see Figure 4).

### 3.3 Correlation results between behavioral performance and fNIRS data

**Figure 5:**
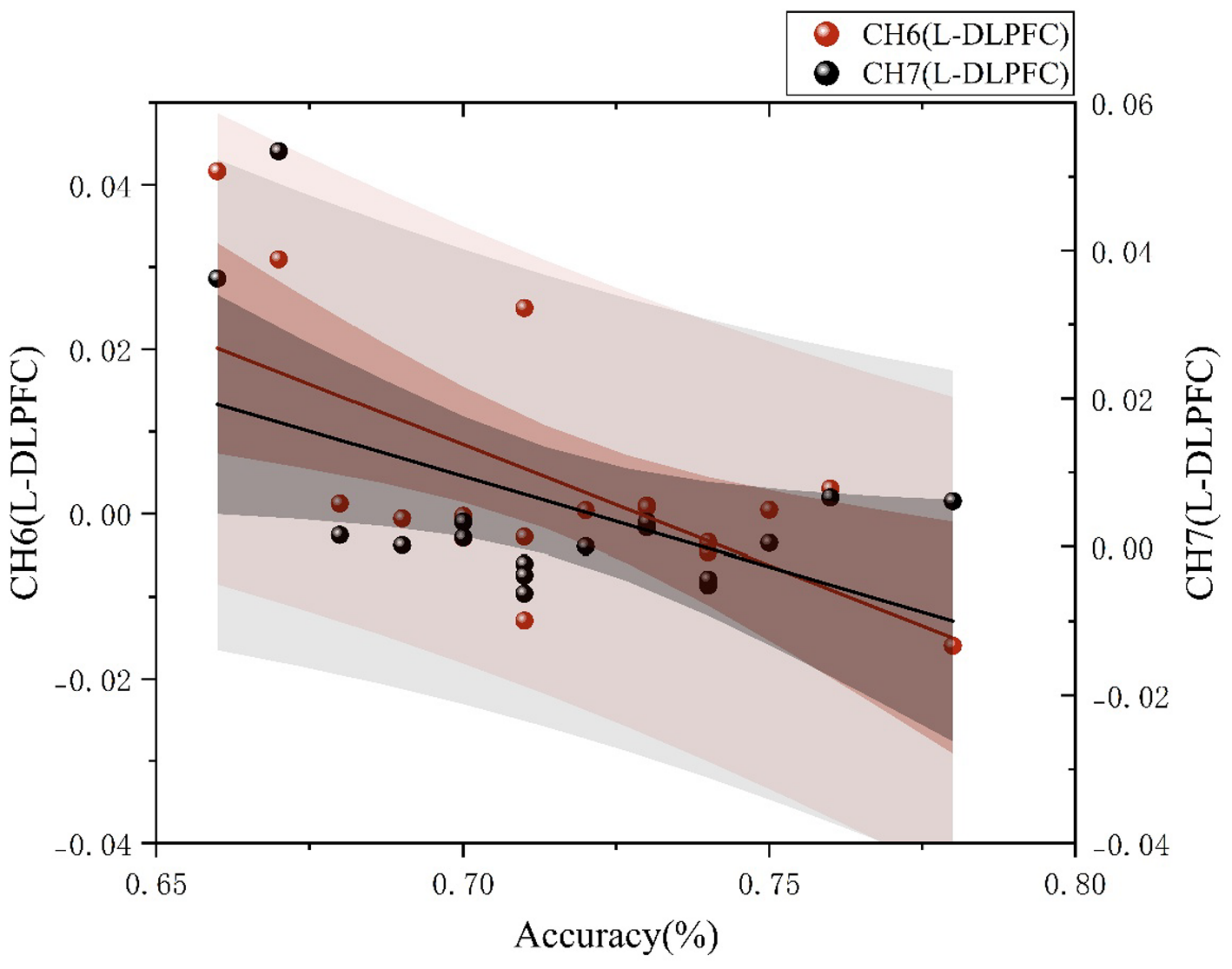
Correlation between accuracy and brain activation values in the expert group during the spatial orientation task.

To further investigate the relationship between behavioral performance and brain activation, Pearson’s correlation analyses were conducted between accuracy and HbO_2_ concentrations in ROIs during the spatial orientation task for the orienteering athletes of different skill levels. The results revealed that, in the expert group, no correlation was found between accuracy and HbO_2_ concentrations during the normal orientation task; during the rotated orientation task, accuracy was negatively correlated with Ch6 (*r* = −0.630, *p* = 0.007) and Ch7 (*r* = −0.503, *p* = 0.039). No significant correlations were found in the novice group (see Figure 5).

## 4 Discussion

The present study investigated behavioral performance and prefrontal cortical activation during map-representation tasks under normal- and rotated-orientation conditions in expert and novice orienteers. The behavioral results showed that rotated orientation impaired task performance, as reflected by lower accuracy and longer reaction times than in the normal-orientation condition. In addition, expert orienteers outperformed novices overall, showing higher accuracy and faster responses. Notably, the reaction-time advantage of experts was particularly evident in the rotated condition, whereas no significant between-group difference was observed in the normal condition. This pattern suggests that expertise is particularly beneficial when task demands increase and when participants must resolve map–environment mismatch.

As a fundamental skill in orienteering, map representation requires individuals to extract relevant map information, transform two-dimensional symbolic input into an internal spatial representation, and match that representation to the real-world environment in order to identify the correct target location (Zhao et al., 2023). This process depends not only on map-reading ability but also on spatial localization, environmental recognition, and the continuous updating of spatial relations (Liu, 2019). Expert orienteers, through prolonged domain-specific training, are likely to develop more efficient and accurate ways of processing map symbols, integrating environmental cues, and establishing map-to-terrain correspondence. This interpretation is consistent with previous findings showing that experts outperform novices in map interpretation, route planning, and spatial-orientation tasks (Guo et al., 2024). In the present study, such expertise-related advantages were reflected in both higher accuracy and faster behavioral responses.

The effect of spatial orientation on performance further suggests that rotated maps impose greater cognitive demands than normally aligned maps. Under rotated-orientation conditions, participants had to resolve the mismatch between the map and the environmental reference frame before identifying the correct target location (Song et al., 2021). This process likely increases the demands on spatial transformation, attentional control, and working-memory updating, thereby prolonging response time and reducing accuracy (Hu Y. et al., 2025). From the perspective of Cognitive Load Theory, these additional processing requirements increase task difficulty and place greater demands on limited cognitive resources (van Nooijen et al., 2024). In the normal-orientation condition, such demands are relatively modest, which may explain why group differences were less pronounced. By contrast, the rotated condition required more complex information integration, thereby magnifying the behavioral advantage of expertise.

The fNIRS findings provide further insight into the neural processes underlying these behavioral differences. Significant hemodynamic changes were observed in the R-VLPFC under the rotated-orientation condition relative to the normal-orientation condition in both experts and novices. This pattern suggests that increased R-VLPFC activation reflects a core cognitive process required by rotated-map matching rather than a compensatory process specific to one group (Cheng et al., 2022; Hu F. et al., 2025). When the map is misaligned with the environmental reference frame, participants must suppress the initially mismatched orientation, re-establish map-to-environment correspondence, and update the spatial representation needed for target localization (Ou et al., 2025). These demands are common to both experts and novices and likely account for the increased R-VLPFC activation observed across groups. Therefore, the R-VLPFC increase in the rotated condition is better interpreted as reflecting shared spatial reorientation and interference-control demands during map processing.

In addition to the common increase in R-VLPFC activation, group differences were observed in other prefrontal subregions. In the map-representation task, experts showed lower activation than novices in the frontal pole area. One possible explanation is the neural efficiency hypothesis, which proposes that individuals with higher levels of expertise can achieve better performance at lower neural costs (Babiloni et al., 2010; Nussbaumer et al., 2015). Previous studies in sports and cognitive neuroscience have reported similar patterns, suggesting that extensive training may reduce the amount of cortical recruitment required to perform demanding tasks (Zhang et al., 2019). In the present study, map representation required recognition, encoding, transformation, and matching of spatial information, all of which imposed substantial executive demands. The lower frontal activation observed in experts may therefore indicate that they completed these operations more efficiently than novices.

At the same time, the lower activation observed in experts should not be interpreted solely as evidence of neural efficiency. Another plausible explanation is that experts rely on different cognitive strategies during map representation (Liu et al., 2024). Long-term orienteering training may promote more automated, holistic, or allocentric modes of spatial processing, whereas novices may depend more on effortful, stepwise, or egocentric processing strategies (Ou et al., 2026). If so, reduced prefrontal activation in experts may reflect not only lower processing cost but also qualitative differences in how spatial information is encoded, transformed, and matched to the environment. Therefore, expertise-related neural differences in this study may be explained by both greater efficiency and differences in strategy use.

This interpretation is particularly relevant to the DLPFC findings. The DLPFC has been widely associated with working-memory maintenance (Barbey et al., 2013), monitoring (Sharp et al., 2004), and executive control under increased task difficulty (Jasinska et al., 2012). In the present study, no clear between-group difference in DLPFC activation was observed in the normal-orientation condition, but novices showed greater DLPFC activation than experts under rotated-orientation conditions. This pattern suggests that novices required greater executive-control and working-memory resources to maintain, manipulate, and verify spatial information when the map orientation was inconsistent with the environmental reference frame (Mariani, 2005; Meneghetti et al., 2022). This interpretation is also consistent with behavioral results, which showed that novices required substantially more time than experts in the rotated condition. In other words, rotated-map processing appears to impose a greater compensatory burden on the DLPFC in novices, whereas experts complete the task with less reliance on additional executive control.

Taken together, these findings suggest a functional distinction between prefrontal subregions during rotated-map processing. Increased R-VLPFC activation in both groups appears to reflect a shared, task-general mechanism of spatial reorientation and interference control. In contrast, stronger activation in the DLPFC and frontal pole in novices likely reflects additional compensatory demands on working memory, attentional monitoring, and executive control. Therefore, expertise in orienteering may be associated not only with superior behavioral performance but also with a reduced reliance on compensatory prefrontal control during spatially demanding map-representation tasks.

The present study did not reveal significant differences in the orbitofrontal area. This null finding suggests that the OFA may play a less prominent role in the specific map-representation and spatial-reorientation demands examined in this study (Epstein et al., 2017) or that its contribution may not have been sufficiently captured under the present task conditions and measurement resolution. Further studies will be needed to clarify whether orbitofrontal involvement emerges under tasks with stronger affective, reward-related, or decision-uncertainty components (Rolls et al., 2020).

### 4.1 Limitations

Several limitations of the present study should be acknowledged. First, the sample size was relatively modest, and the two groups were unequal in size, which may have limited statistical power and the generalizability of the findings. In addition, although the rotated-orientation condition involved multiple rotation angles, these angles were analyzed as a single condition in the present study. As a result, potential differences among specific rotation angles could not be examined. Moreover, because the study adopted a cross-sectional expert–novice design, the findings should not be interpreted as direct evidence of a causal effect of training.

Second, several methodological limitations related to fNIRS should be considered. fNIRS primarily measures hemodynamic changes in cortical surface regions and cannot directly capture activity in deeper structures, such as the hippocampus and other medial temporal areas that are known to contribute to spatial memory and navigation. Accordingly, the present findings should be interpreted specifically as evidence of prefrontal cortical involvement rather than as reflecting the full neural network underlying spatial orientation. In addition, although both HbO_2_ and HbR signals were derived during preprocessing, the present study focused on HbO_2_ as the primary outcome measure. While this approach is common in task-based fNIRS research, it limits the physiological completeness of the reported results.

Third, statistical results should also be interpreted with appropriate caution. Although multiple comparisons across channels were controlled using the false discovery rate (FDR) procedure, channel-wise analyses of a relatively small sample may still yield unstable estimates. A related limitation concerns the number and structure of the fNIRS trials. The formal experiment included only 12 trials per condition, with 4 trials in each block. Although this design was intended to balance task demands, participant fatigue, and hemodynamic recovery, the number of trials per condition was relatively limited for a cognitively demanding fNIRS paradigm involving map representation, spatial transformation, and executive control. In addition, the relatively short trial sequence within each block may have reduced the stability of sustained hemodynamic responses. Future studies should therefore include larger samples, increase the number of valid trials per condition, and further optimize block length, temporal structure, and rotation-angle manipulation to improve signal reliability, statistical power, and the interpretability of the findings.

## 5 Conclusion

This study used fNIRS to investigate orienteers’ behavioral performance and changes in blood oxygen concentration of the prefrontal cortex (PFC) during different spatial orientation tasks. The findings are as follows: (1) Map rotation degraded behavioral performance; however, expert orienteers still maintained better performance than novices, demonstrating sport-specific expertise. (2) Rotated orientation resulted in distinct hemodynamic modulation in the prefrontal cortex, with the left dorsolateral prefrontal cortex, right ventrolateral prefrontal cortex, and the frontal pole exhibiting particularly pronounced activation. (3) Long-term specialized orienteering training endowed expert orienteers with lower neural activation during spatial tasks. These findings offer a theoretical foundation for designing scientifically validated training programs to optimize wayfinding and navigation skills in orienteering.

## Data Availability

The raw data supporting the conclusions of this article will be made available by the authors, without undue reservation.
